# Magnetic resonance imaging of the knee for chronological age estimation—a systematic review

**DOI:** 10.1007/s00330-023-09546-8

**Published:** 2023-04-12

**Authors:** Lil-Sofie Ording Muller, Jan Adolfsson, Lisa Forsberg, Johan Bring, Jovanna Dahlgren, Helena Domeij, Carl Gornitzki, Emma Wernersson, Jenny Odeberg

**Affiliations:** 1 Division of Radiology and Nuclear Medicine, Department of Paediatric Radiology, Oslo University Hospital, Rikshospitalet, Oslo Norway; 2 Swedish Agency for Health Technology Assessment and Assessment of Social Services, Stockholm, Sweden; 3 Department of Clinical Science, Intervention and Technology-CLINTEC, Karolinska Institutet, Stockholm, Sweden; 4 Statisticon AB, Uppsala, Sweden; 5 Department of Pediatrics, Sahlgrenska Academy at the University of Gothenburg, Gothenburg, Sweden

**Keywords:** Systematic review, Magnetic resonance imaging, Knee joint, Age determination by skeleton, Predictive value of tests

## Abstract

**Introduction:**

Radiographs of the hand and teeth are frequently used for medical age assessment, as skeletal and dental maturation correlates with chronological age. These methods have been criticized for their lack of precision, and magnetic resonance imaging (MRI) of the knee has been proposed as a more accurate method. The aim of this systematic review is to explore the scientific and statistical evidence for medical age estimation based on skeletal maturation as assessed by MRI of the knee.

**Materials and methods:**

A systematic review was conducted that included studies published before April 2021 on living individuals between 8 and 30 years old, with presumptively healthy knees for whom the ossification stages had been evaluated using MRI. The correlation between “mature knee” and chronological age and the risk of misclassifying a child as an adult and vice versa was calculated.

**Results:**

We found a considerable heterogeneity in the published studies —in terms of study population, MRI protocols, and grading systems used. There is a wide variation in the correlation between maturation stage and chronological age.

**Conclusion:**

Data from published literature is deemed too heterogenous to support the use of MRI of the knee for chronological age determination. Further, it is not possible to assess the sensitivity, specificity, negative predictive value, or positive predictive value for the ability of MRI to determine whether a person is over or under 18 years old.

**Key Points:**

• *There is an insufficient scientific basis for the use of magnetic resonance imaging of the knee in age determination by skeleton.*

• *It is not possible to assess the predictive value of MRI of the knee to determine whether a person is over or under 18 years of age.*

**Supplementary Information:**

The online version contains supplementary material available at 10.1007/s00330-023-09546-8.

## Introduction

Determining a person’s age is important for many legal processes, e.g., regarding child labor, sexual assault, prostitution, and sometimes for elite athletes, but is particularly relevant in the asylum procedure. In most European countries, the cut-off age of minority is 18 years of age [1, 2]. Individuals below the age of 18 are entitled to have their legal rights as minors respected in accordance with national and international laws and regulations [3]. Medical tests to assess a person’s psychological and physiological development level have been used as methods for chronological age assessment, particularly in young unaccompanied asylum seekers when their date of birth is unknown, or when they lack documents to confirm their age [4]. There is a correlation between skeletal and dental development and chronological age; hence, methods for the assessment of skeletal maturation based on radiographs of the hand and maturation of the teeth are frequently used to assign a chronological age to an individual. However, those methods have been criticized for their lack of precision [5, 6].

Magnetic resonance imaging (MRI) has been proposed as a more advanced imaging technique for the evaluation of skeletal maturation. MRI is radiation free and can be applied to several bones, e.g., the clavicle and the hand. MRI of the knee is a method which in the recent decade has been proposed to potentially provide a more accurate method for chronological age assessment than traditional radiographic methods [7–9].

Grading of skeletal maturation of the knee is based on the appearances of the physeal line, or physis, which is the site of growth in a long bone. The physis is a fine structure consisting of mesenchymal cells in different maturation stages. The physis becomes thinner and thinner throughout the process of skeletal maturation, before it disappears and endochondral ossification ceases. There are six different grading systems for skeletal maturation of the knee assessed by MRI: (1) Schmeling and Kellinghaus, (2) Vieth, (3) Dedouit, (4) Dedouit, Kellinghaus, and Schmeling, modified version, (5) Jopp and (6) Schmeling. All grading systems classify the maturation into exclusive stages based on the characterization and delineation of the thin physeal line (Tables 1 and 2).

**Table 1:** Descriptive summary of the included studies

Study	Land	MRI classification system (no of stages)	Study design (no participants)	% assessed by 2 readers	MRI- protocol Field strength, Plane, Weighting	Resolution
Alatas [10]	Turkey	Vieth (2–6)	Retrospective (709)	21 %	1.5 T Coronal PD, T1 tse	2 x 4 x 3.5 mm
Altinsoy [11]	Turkey	Dedouit (1–5)	Retrospective (472)	100 %	1.5 T Coronal PD	0.9 x 1.6 x 4.5 mm
Auf der Mauer [12]	Germany	Jopp (1–3)	Prospective longitudinal (40)	100 %	3 T Coronal T1 sense	0.1875 × 0.1875 mm x 2
Daghighi [13]	Iran	Schmeling (1–5)	Retrospective (193)	100 %	1.5 T Sagittal coronal PD fs, T2 tse	Unclear
Dedouit [14]	France	Dedouit (1–5)	Retrospective (290)	100 %	1.5 T Sagittal coronal PD	Unclear
Ekizoglu [15]	Turkey	Schmeling, Kellinghaus (1–5)	Retrospective (649)	100 %	1.5 T Sagittal T1 tse	0.35 x 0.35 x 1.5 mm
Gurses [16]	Turkey	Vieth (2–6)	Retrospective (598)	100 %	1.5 T Coronal PD, T1 tse	0.78 x 1.56 x 3.5 mm
Kramer [17]	Germany	Schmeling, Kellinghaus (1–5)	Retrospective (290)	10 %	3 T Sagittal T1 tse	0.4 × 0.4 × 3.0 mm
Kvist [18]	Sweden	Dedouit, Kellinghaus Schmeling (1–5)	Prospective (395)	100 %	1.5 T Sagittal coronal T1 tse, cartilage	0.62 x 0.62 x 3 mm
Margalit [19]	USA	Dedouit (1–5)	Retrospective (165)	100 %	1.5 and 3 T Fse, «inter-mediate»	Unclear
Ottow [20]	Germany	Schmeling, Kellinghaus (1–5)	Prospective (658)	17 %	3 T T1 tse	0.6 × 0.77 × 3 mm
Uygun [21]	Turkey	Dedouit (1–5)	Retrospective (489)	Unclear	1.5 T PD	Unclear
Vieth [22]	Germany	Vieth (2–6)	Prospective	14 %	3 T T1, T2 spir	0.6 × 0.76 × 3 mm

**Table 2:** The stages used in respective MRI grading system that represent a “mature knee”

MRI grading system	Stage that represents a mature knee	Studies
Schmeling and Kellinghaus	Stage 4 (out of 5)	Kramer [17] Ekizoglu [15] Ottow [20]
Vieth	Stage 5 and 6 (6 stages in total)	Vieth [22] Gurses [16] Alatas [10]
Dedouit	Stage 4 and 5 (5 stages in total)	Dedouit [14] Altinsoy [11] Uygun [21]
Dedouit, Kellinghaus and Schmeling, modified version	Stage 5 (5 stages in total)	Kvist [18]

The aim of this systematic review is to explore the scientific evidence for medical age estimation based on skeletal maturation as assessed by MRI of the knee. We also wanted to explore the likelihood for a minor to be misclassified as an adult, or vice versa, for an adult to be misclassified as a minor, when MRI of the knee is used for chronological age estimation in a forensic setting.

## Materials and methods

### Protocol and registration

This systematic review was conducted at the Swedish Agency for Health Technology Assessment and Assessment of Social Services (SBU), an assignment by the Ministry of Health and Social Affairs in Sweden, and published in Swedish in October 2021 [23]. SBU uses a peer-reviewed protocol for systematic reviews. The systematic review process follows the general concepts covered by Preferred Reporting Items for Systematic Reviews and Meta-analyses, PRISMA [24].

### Eligibility criteria

A study was considered eligible if it reported data for living study participants between the ages of 8 and 30 years with no pathological problems of the knee or ankle (population) for whom the ossification stages of the knee (distal femur) had been evaluated using MRI (index test). The chronological age was known through records (reference test), and the diagnostic accuracy (outcome) was reported as sensitivity/specificity or by a correlation of age and ossification stage. Only cross-sectional studies and longitudinal studies written in English, German, or any Scandinavian language were included.

### Literature search

A systematic literature search was conducted by an information specialist in the following databases: Cochrane Library (Wiley), Embase (Elsevier), Medline (OvidSP), Epistemonikos, KSR Evidence, and International HTA Database. The search strategy was developed and executed in close collaboration with the co-authors LSOM (radiologist) and JD (pediatrician).

A systematic review by Ding et al [25] was used as a starting point for the literature search. Our search was thus limited to studies published between January 2017 and March 2021; all studies included in the systematic review by Ding et al were evaluated for eligibility (see section “Study selection”).

In addition, a reference and citation search of the included studies was performed in the database Scopus (Elsevier). The complete search strategy is provided in Supplement 1.

### Study selection

Two reviewers independently screened the titles and abstracts identified by the literature search strategy. All studies of potential relevance according to the inclusion criteria were obtained in full text, and two reviewers independently assessed them for inclusion. In addition, the articles included in the systematic review by Ding et al [25] were screened for relevance. Any disagreement was resolved by discussion. Excluded studies are shown in Supplement 2.

### Risk of bias in individual studies

Quality assessment (risk of bias) of the included studies, both from the literature search and the systematic review by Ding et al [25], was performed by two independent reviewers using a modified version of Quality Assessment of Diagnostic Accuracy Studies (QUADAS)-2 (Supplement 3). Any disagreement was resolved by discussion. Each study was rated as having low, moderate, or high risk of bias. Studies with high risk of bias were not included in the analysis.

### Data collection process

Data was extracted and tabulated from each included study with low or moderate risk of bias by one reviewer. The extracted data was audited by a second reviewer. The extracted data were study design; how the index test, reference test, and outcome measured; population; and setting (Supplement 4).

### Method of analysis

Data from each study was presented as a correlation between “mature knee” and age. In the studies where the number of subjects in each stage was presented for each age group, no recalculation was required [11, 14, 18, 21]. When data was presented using descriptive statistics regarding the age distribution conditional on being in a specific stage, i.e., mean age ± standard deviation (SD) for each stage, recalculation was performed. The mathematical method used for recalculation was based on a method described by Mostad et al [26] and Bleka et al [27], with the modification that we relaxed the assumption about normality and instead used the information about the exact age distribution of the subjects included in the study [23].

### Rating the certainty of the evidence

The certainty in the estimated prevalence rates was supposed to be assessed using grading of recommendations assessment, development, and evaluation (GRADE) [28]. However, since the studies were too heterogenous to perform a meta-analysis, or even for a meaningful narrative review, we decided to refrain from a GRADE assessment.

## Results

**Fig. 1:**
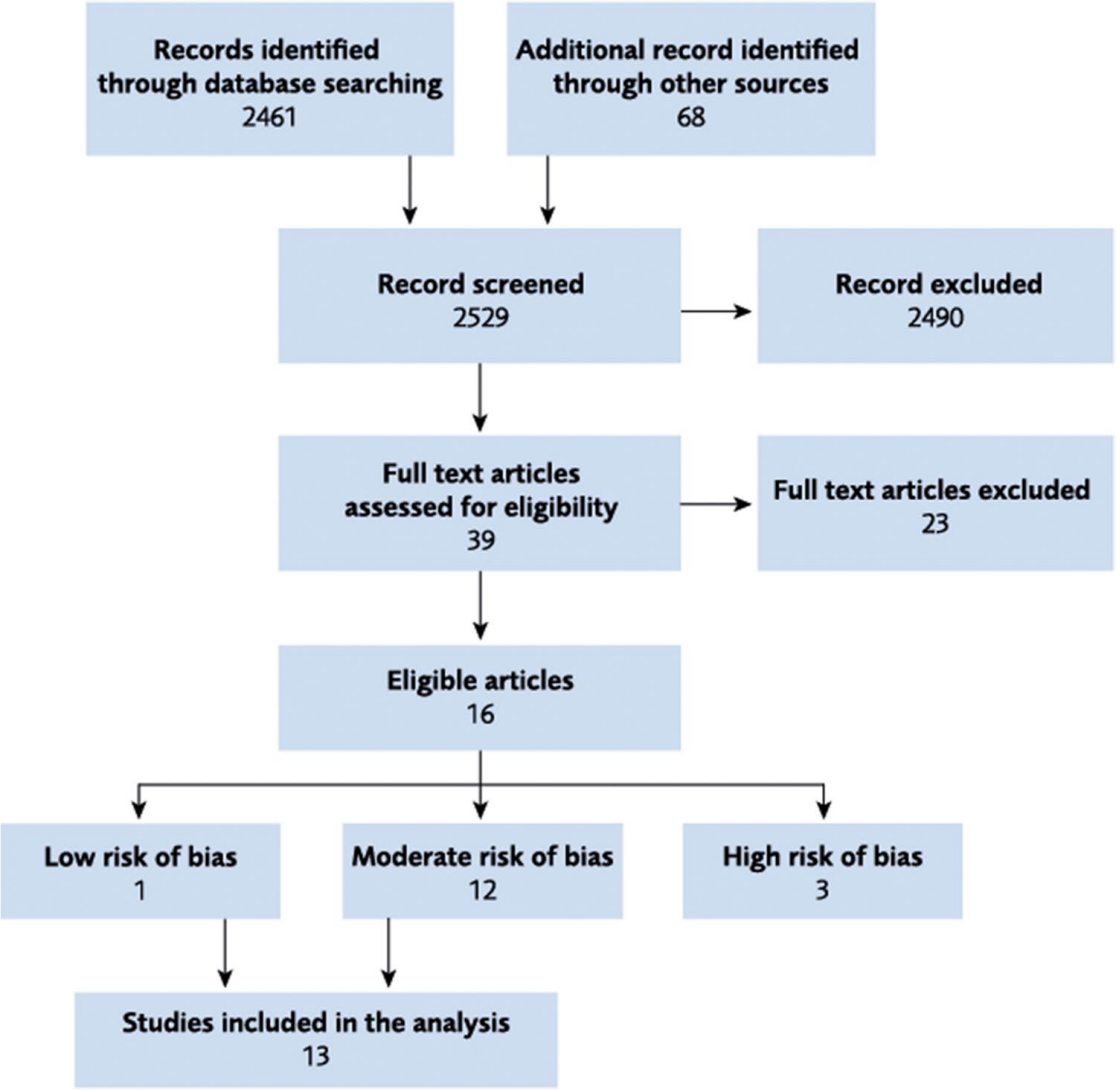
Flow chart of literature selection

The literature search yielded 2529 references to be screened (see Fig. 1). Of these, 39 were reviewed in full text [10–22, 25, 26, 29–52]. A total of 16 articles were considered eligible since they met the inclusion and exclusion criteria. Of these, 13 had low or moderate risk of bias and are included in the analysis [10–22]. The risk of bias chart is shown in Supplement 5. Three articles had high risk of bias: one due to an inadequate description of the MRI method, one lacked complete information regarding birth date, and one due to several limitations. See Supplement 2 for the list of excluded studies. The characteristics of the included studies are presented in Supplement 4.

### Study characteristics

Out of the 13 studies, 9 were retrospective [10, 11, 13–17, 19, 21] and 4 prospective [12, 18, 20, 22]. The studies were from Iran [13], Turkey [10, 11, 15, 16, 21], Germany [12, 17, 20, 22], Sweden [18], France [14], and USA [19]. The study population consisted of various subjects such as patients who underwent MRI for assessment of traumatic or degenerative changes to the knee joint [10, 11, 14, 16, 17, 19, 21], healthy volunteers (4 studies) [12, 18, 20, 22], and individuals assessed due to legal reasons (1 study) [13]. The information provided regarding the study population was sparse in most papers.

The studies used 6 different grading systems for evaluation of maturation stage of the knee on MRI: Schmeling and Kellinghaus [15, 17, 20], Vieth [10, 16, 22], Dedouit [11, 14, 19, 21], Dedouit and Kellinghaus (modified version of Schmeling) [18], Jopp [12], and Schmeling [13] (Table 1). The MRI grading systems differ both in terms of the number and the definition of stages of maturation. Stages varied between 3 and 5, with up to 11 substages. The stages were described with varying degrees of detail in the original publications, and there was a mix of quantitative and qualitative criteria. Therefore, the stages in the different grading systems were overlapping and could not be pooled. The MRI protocols used varied between the studies (see Table 1).

In 8 studies, two readers had reviewed all images, but in 4 studies, only 10–21% of the images were reviewed by two readers, and in one study, the information was unclear (Table 1).

### The risk for misclassification

The studies were deemed too heterogeneous with regard to MRI protocols, grading system, and population; therefore, a meta-analysis was not performed (Table 1 and Supplement 4). Even in the studies using the same grading system, the population and MRI protocol were considered too heterogeneous to allow a meta-analysis. We therefore present the results from each study individually. The results are presented separately for males and females. We present the results for the most relevant classification stages for age determination, where individuals both over and under 18 years are represented (Table 2).

Of the 13 studies, 4 presented data as stage per age [11, 14, 18, 21] and no recalculations had to be performed. For the remaining nine studies [10, 12, 13, 15–17, 19, 20, 22], recalculation was performed for six of them [10, 15–17, 20, 22]. Two studies could not be included in the analysis since they lacked information about minimum and maximum age which is required for the recalculations [13, 19], and one study had only sufficient information for 6 participants [12].

**Fig. 2:**
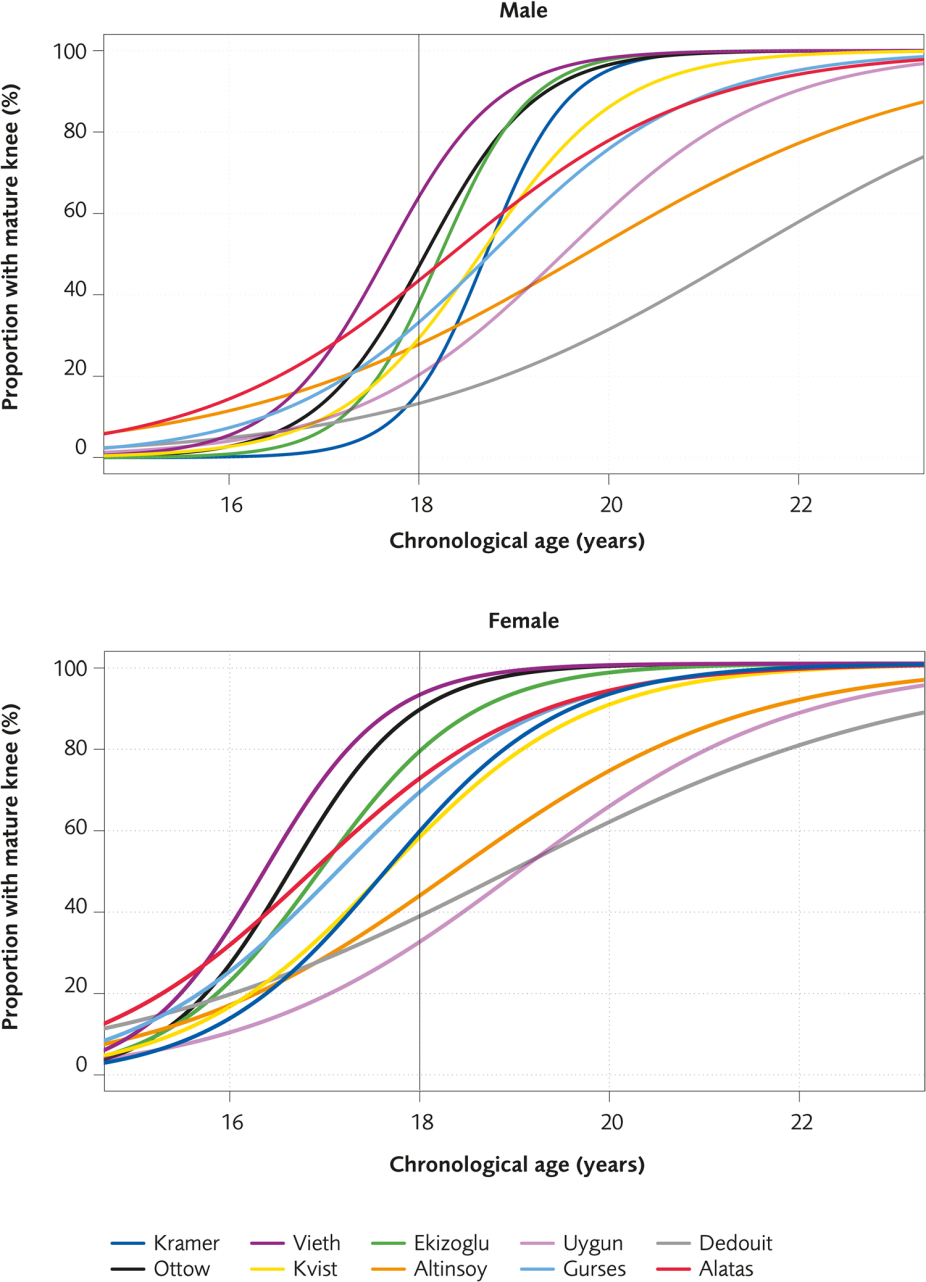
The proportion of males or females (%) for the respective MRI grading system with a “mature” knee

In Fig. 2, the proportion of males or females with a mature knee per age (interval 15–23 years) is presented. The curves are estimated by using a logistic regression function, $(p(x)=1/1+e^{-(\beta_0+\beta_1X)}$, with age as the independent variable and mature/not-mature as the dependent variable. The model estimates the probability of having a mature knee as a function of age.

The data show clearly how the results differ between the studies. For example, the maturation of the knee for males seems to appear later in the studies by Kramer et al [17] and Dedouit et al [14] than in the studies by Vieth and Ottow et al [20, 22].

**Fig. 3:**
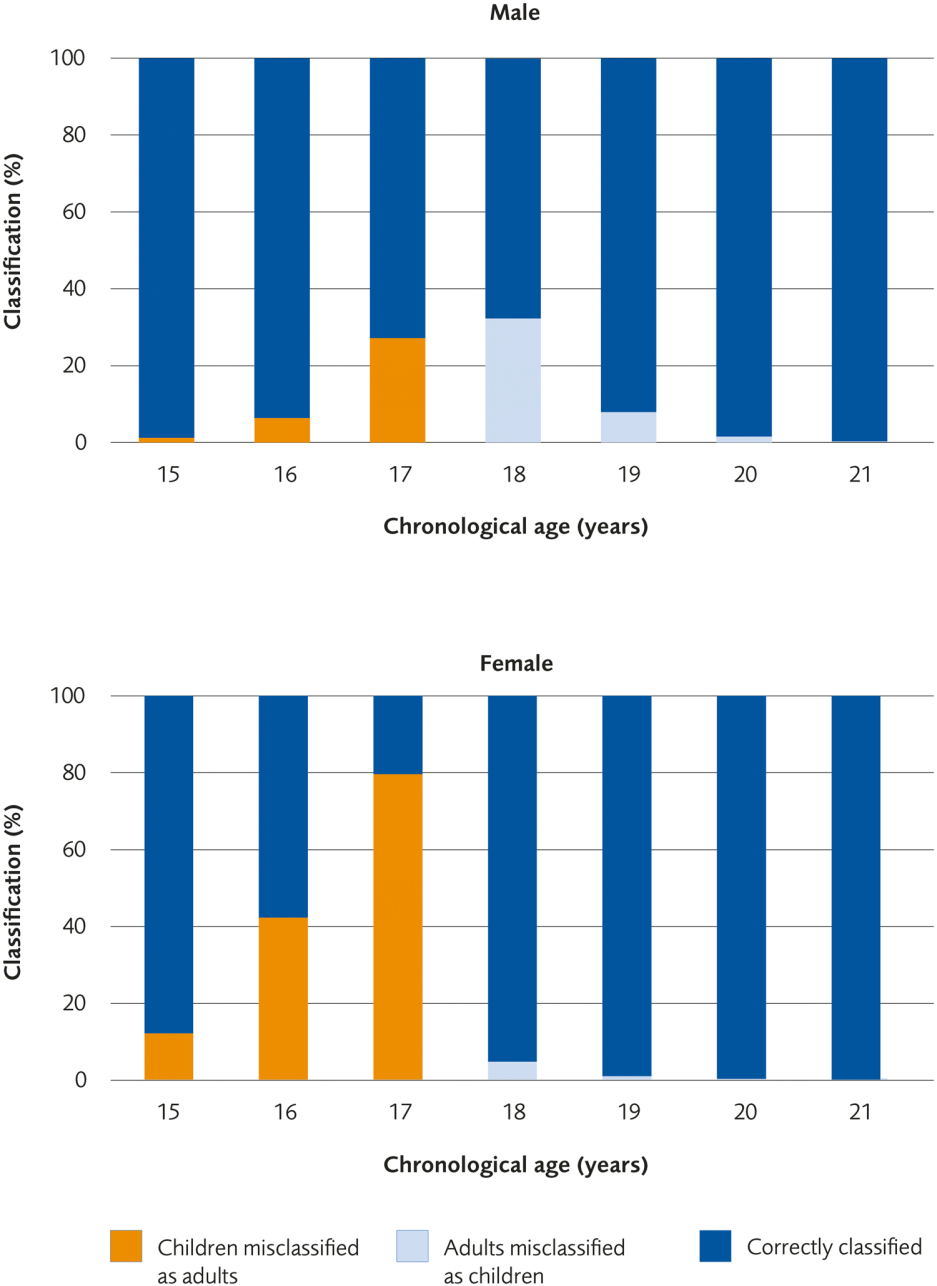
Correct classification and misclassification per age (15–21 years of age) subdivided for males and females (%) based on the study by Ottow et al

We calculated the risk for misclassification, i.e., the risk that a minor (an individual 17 years or younger) would be misclassified as an adult (an individual 18 years or older) or that an adult would be misclassified as a minor, in the age interval of 15 to 21 years for each of the studies. Figure 3 illustrates the results based on data from the study by Ottow et al [20]. In Table 3, the results for all grading systems and all studies are presented. The risk of misclassification varies between studies (Table 3). The risk for misclassification is generally much higher in females than in males until the age of 17, at which point males are more likely to be misclassified. For females, where the knee matures earlier, the highest risk for misclassification is between 16 and 18 years of age. For males, the risk is the highest between the ages of 17 and 18 years.

**Table 3:** The risk for males and females to be misclassified, presented per age group, study and grading system

	Grading system							
Per age	Schmeling and Kellinghaus		Vieth		Dedouit		Dedouit, Kellinghaus and Schmeling, modified version	
	Male	Female	Male	Female	Male	Female	Male	Female
15-years	0% [17] 1% [15] 1% [20]	8% [17] 12% [15] 12% [20]	2% [22] 5% [16] 10% [10]	18% [22] 16% [16] 21% [10]	4% [14] 9% [11] 3%[21]	16% [14] 12% [11] 7% [21]	1% [18]	10% [18]
16-years	1% [17] 3% [15] 6% [20]	21% [17] 34% [15] 42% [20]	12% [22] 11%[16] 20% [10]	52% [22] 34% [16] 41% [10]	6% [14] 15% [11] 7% [21]	24% [14] 22% [11] 15% [21]	5% [18]	24% [18]
17-years	6% [17] 19% [15] 27% [20]	47% [17] 67% [15] 80% [20]	43% [22] 24% [16] 35% [10]	85% [22] 59% [16] 64% [10]	11% [14] 23% [11] 15% [21]	34% [14] 37% [11] 26% [21]	17% [18]	47% [18]
18-years	62% [17] 38% [15] 32% [20]	26% [17] 11% [15] 5% [20]	19% [22] 56% [16] 47% [10]	4% [22] 20% [16] 18% [10]	83% [14] 66% [11] 72% [21]	54% [14] 46% [11] 57% [21]	55% [18]	28% [18]
19-years	14% [17] 8% [15] 8% [20]	10% [17] 3% [15] 1% [20]	4% [22] 34% [16] 29% [10]	1% [22] 8% [16] 8% [10]	74% [14] 53% [11] 51% [21]	41% [14] 29% [11] 39% [21]	24% [18]	12% [18]
20-years	2% [17] 1% [15] 2% [20]	3% [17] 1% [15] 0 % [20]	1% [22] 17% [16] 16% [10]	0% [22] 3% [16] 3% [10]	62% [14] 40% [11] 31% [21]	30%[14] 17% [11] 23% [21]	8% [18]	5% [18]
21-years	0% [17] 0% [15] 0% [20]	1% [17] 0% [15] 0% [20]	0% [22] 7% [16] 8% [10]	0% [22] 1% [16] 1% [10]	49% [14] 28% [11] 16 % [21]	20% [14] 9% [11] 12% [21]	2% [18]	2% [18]

### Positive predictive value, negative predictive value, sensitivity, and specificity

To be able to calculate positive predictive value (PPV), negative predictive value (NPV), specificity, and sensitivity, the frequency/ratio of mature and immature knee per age, as well as the age distribution of the population tested, needs to be known. The risk of misclassification depends on the actual age distribution of those tested. For example, if many individuals are tested in the age range 17 to 18 years, the risk of misclassification will be high. If the age range is widened, the proportion of errors will be lower.

To illustrate this issue, we have performed calculations on five hypothetical populations, each with a different age distribution (Table 4).

**Table 4:** Number of individuals per age in five different hypothetical populations

Population	15 years of age	16 years of age	17 years of age	18 years of age	19 years of age	20 years of age
17–18	0	0	*100*	*100*	0	0
16–19	0	*100*	*100*	*100*	*100*	0
15–20	*100*	*100*	*100*	*100*	*100*	*100*
15–18	*100*	*100*	*100*	*100*	0	0
17–20	0	0	*100*	*100*	*100*	*100*

**Fig. 4:**
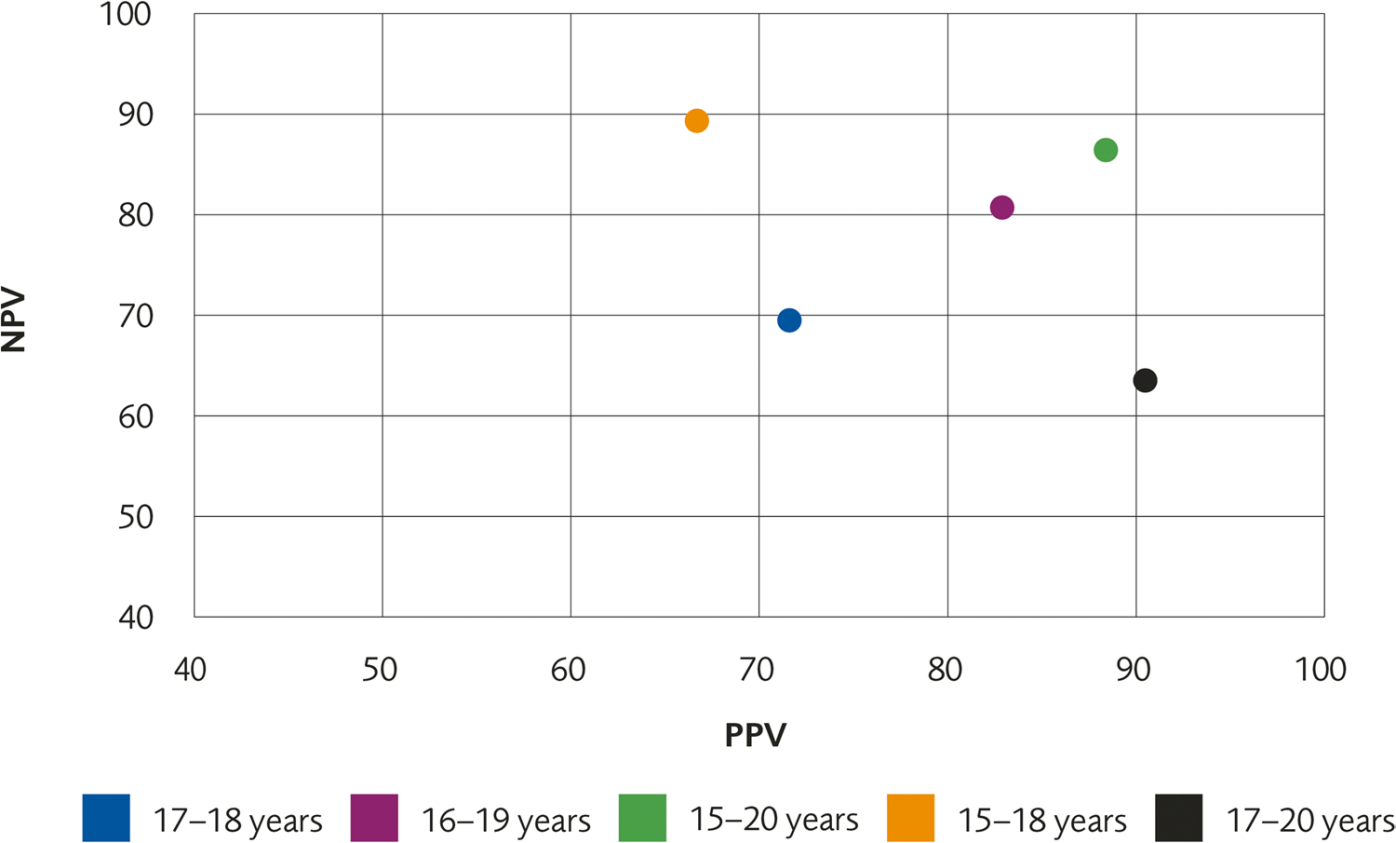
Illustration of how PPV and NPV vary depending on the age composition of the populations tested. Based on data from Ottow et al (male)

Using data from the study by Ottow et al (male) [20], we illustrate how the same datapoints give rise to different predictive values in response to variation in the age distribution of the population tested (Fig. 4). This is further elaborated in Supplement 6 where predictive values using datapoints from other included studies on the same five hypothetical populations are presented. The calculations show that NPV, PPV, sensitivity, and specificity for MRI of the knee as a test to determine whether a person is over or under 18 years old cannot be calculated in individuals between 15 and 21 years of age unless the exact *age composition* of the group is known.

## Discussion

This systematic review shows that the proportion of individuals per age group deemed to have a mature knee on MRI varies considerably between the included studies. Women’s knees seem to mature earlier than men’s knees; however, the magnitude of the difference was not consistent between studies. The calculated risk for misclassification for each study also varied because it depends on the dataset from which the calculations are based. The variation in results of maturation according to age can be explained by the different grading systems, MRI protocols, and study populations in the published studies.

Further, we illustrate how calculating the risk of misclassification for the purpose of forensic age determination is paradoxical, as that risk can only be calculated in a population with a known age distribution. This fundamental statistical limitation cannot be overcome, no matter how advanced the measurement techniques are, or how well future studies are conducted.

Maturation of the knee is influenced by individual factors such as genetics, medical conditions, and nutrition [53]. This causes a wide, and somewhat unpredictable, variation in skeletal maturation rates. Since these factors naturally vary between individuals, the correlation between chronological age and bone maturation is influenced by the characteristics of the study population.

MRI is probably the modality within the field of radiology which is the most difficult to standardize, both in terms of image acquisition and image reading [54, 55]. Therefore, results from studies performed in different institutions, on different MRI machines, with different readers, different image scan parameters, and with different scoring systems, are not directly comparable, and to do so will result in substantial variability. For instance, Kvist et al [18] demonstrated that the use of different weightings applied on the same individuals influenced the grading of physeal maturation. Objective assessment of MRI signal is difficult because readers tend to perceive the same image intensity differently, depending on surrounding background intensities [56]. Reader experience and calibration of the reading structure also influence the interpretation of the images [18].

One strength of this systematic review is the strict adherence to international standards for systematic reviews. Studies were identified and selected according to the PRISMA statement which is internationally regarded as state of the art for performing and reporting systematic reviews (see Supplements 1–5). Another strength is our multidisciplinary approach where information specialists and statisticians worked in close collaboration with clinical experts, including pediatricians and a pediatric radiologist.

The main limitation of this systematic review is that relatively few studies were identified. Larger and more comparable studies may be able to show a stronger trend in terms of the proportion of individuals per age group deemed to have a mature knee on MRI. Still, we will not be able to able to predict the risk of misclassification in individuals with an unknown age due to the statistical paradox described above.

Forensic age estimations can have huge legal implications for the individual being evaluated, as well as for countries and authorities who use such methods. Therefore, the methods used should be reliable and reproducible and the statistical calculations of probability must exactly fit the population to which it is applied.

In conclusion, there is a considerable heterogeneity in the published studies on forensic age assessment based on MRI of the knee. Therefore, neither a meta-analysis nor a meaningful narrative review could be performed. Furthermore, the actual risk of misclassifying a minor as an adult and vice versa can never be calculated in a group of individuals with unknown age distribution.

## Supplementary Information

Below is the link to the electronic supplementary material.

Supplementary file1 (PDF 2.15 mb)

## Abbreviations

GRADE: Grading of recommendations assessment, development and evaluation
MRI: Magnetic resonance imaging
NPV: Negative predictive value
PPV: Positive predictive value
PRISMA: Preferred Reporting Items for Systematic Reviews and Meta-Analyses
QUADAS: Quality Assessment of Diagnostic Accuracy Studies
SBU: The Swedish Agency for Health Technology Assessment and Assessment of Social Services
SD: Standard deviation

## Declarations

### Guarantor

The scientific guarantors of this publication are Jenny Odeberg and Lil-Sofie Ording Müller.

### Statistics and biometry

One of the authors (Johan Bring) has significant statistical expertise.

### Study subjects or cohorts overlap

A commissioned online report, written in Swedish, published in October 2021 {SBU, 2021 #97}.

### Methodology

• multicenter study
