# Supplementary material for: Magnetic resonance imaging of the knee for chronological age estimation—a systematic review
Source: Eur Radiol. 2023 Apr 12;33(8):5258–68. doi: 10.1007/s00330-023-09546-8 (PMC10326106; doi:10.1007/s00330-023-09546-8)
Supplement: Supplementary file 1 — Supplementary file1 (PDF 2.15 mb) [file 330_2023_9546_MOESM1_ESM.pdf]

Supplement 1 Search strategies

Cochrane Library via Wiley 13 March 2021 (CDSR, Cochrane Protocols & CENTRAL)

Search terms			Items found
**Age assessment**			
1.	2.	((age or matur* or ossification or year*) NEAR/3 (assess* or chronologic* or determ* or estimat* or evaluat* or examin* or forensic or ident* or legal or measur* or predict* or verif*)):ti,ab,kw	33,728
3.		((epiphys* or "growth plate*") AND (adolescen* or age or child* or matur* or ossification or year* or "young adult*")):ti,ab,kw	197
4.		*1 OR 2*	*33,904*
**Knee**			
5.		(femur or femoral or knee* or tibia*):ti,ab,kw	47,628
**MRI**			
6.		("magnetic resonance imag*" or mr or mri or mris or nmr or radiolog* or radiograp*):ti,ab,kw	71,218
**Combined sets**			
7.		*3 AND 4 AND 5 with Publication Year from 2017 to 2021, in Trials*	***175***
8.			
**Final result**			
**9.**			CENTRAL/175

The final search result, usually found at the end of the documentation, forms the list of abstracts.

:ti,ab,kw = Title, abstract or keyword; ***** = Truncation; " " = Citation Marks; searches for an exact phrase; **CDSR** = Cochrane Database of Systematic Review; **Cochrane Protocols** = Protocols of systematic reviews registered in Cochrane Library; **CENTRAL** = Cochrane Central Register of Controlled Trials, “trial

Embase via Elsevier 13 March 2021

Search terms		Items found
**Age assessment**		
1.	'age determination'/exp	7,157
2.	'forensic anthropology'/de	945
3.	'epiphysis'/exp AND ('adolescent'/de OR 'young adult'/de)	1,598
4.	((age OR matur* OR ossification OR year*) NEAR/3 (assess* OR chronologic* OR determ* OR estimat* OR evaluat* OR examin* OR forensic OR ident* OR legal OR measur* OR predict* OR verif*)):ti,ab,kw	390,674
5.	(epiphys*:ti,ab,kw OR 'growth plate*':ti,ab,kw) AND (adolescen*:ti,ab,kw OR age:ti,ab,kw OR child*:ti,ab,kw OR matur*:ti,ab,kw OR ossification:ti,ab,kw OR year*:ti,ab,kw OR 'young adult*':ti,ab,kw)	14,541
6.	*1 OR 2 OR 3 OR 4 OR 5*	408,413
**Knee**		
7.	'knee'/de	72,652
8.	'tibia'/exp	50,583
9.	'femur'/exp	99,787
10.	femur:ti,ab,kw OR femoral:ti,ab,kw OR knee*:ti,ab,kw OR tibia*:ti,ab,kw	484,203
11.	*7 OR 8 OR 9 OR 10*	515,789
**MRI**		
12.	'nuclear magnetic resonance imaging'/de	827,865
13.	'radiography'/de	279,653
14.	'magnetic resonance imag*':ti,ab,kw OR mr:ti,ab,kw OR mri:ti,ab,kw OR mris:ti,ab,kw OR nmr:ti,ab,kw OR radiolog*:ti,ab,kw OR radiograp*:ti,ab,kw	1,524,715
15.	*12 OR 13 OR 14*	1,995,377
**Combined sets**		
16.	*6 AND 11 AND 15*	6,454
17.	*#16 AND (2017:py OR 2018:py OR 2019:py OR 2020:py OR 2021:py)*	*1,717*
**Final result**		
**18.**		**1,717**

The final search result, usually found at the end of the documentation, forms the list of abstracts.

/de = Term from the EMTREE controlled vocabulary; **/exp** = Includes terms found below this term in the EMTREE hierarchy; **:ti,ab,kw** = Title, abstract or keyword; ***** = Truncation; **' '** = Citation Marks, searches for an exact phrase; **NEAR/n** = Requests terms that are within 'n' words of each other in either direction

Medline via OvidSP 13 March 2021

Search terms		Items found
**Age assessment**		
1.	Age Determination by Skeleton/	4,522
2.	Forensic Anthropology/	3,456
3.	Age Factors/	460,586
4.	exp Epiphyses/ and (Adolescent/ or Young Adult/)	2,470
5.	((age or matur* or ossification or year*) adj3 (assess* or chronologic* or determ* or estimat* or evaluat* or examin* or forensic or ident* or legal or measur* or predict* or verif*)).ti,ab,kf.	262,304
6.	((epiphys* or growth plate*) and (adolescen* or age or child* or matur* or ossification or year* or young adult*)).ti,ab,kf.	11,304
7.	*1 OR 2 OR 3 OR 4 OR 5 OR 6*	
**Knee**		
8.	Knee/	14,687
9.	exp Knee Joint/	62,570
10.	Tibia/	35,866
11.	Femur/	42,511
12.	(femur or femoral or knee* or tibia*).ti,ab,kf.	362,387
13.	*8 OR 9 OR 10 OR 11 OR 12*	392,387
**MRI**		
14.	Magnetic Resonance Imaging/	417,253
15.	Radiography/	320,591
16.	(magnetic resonance imag* or mr or mri or mris or nmr or radiolog* or radiograp*).ti,ab,kf.	1,124,815
17.	*14 OR 15 OR 16*	1,472,256
**Combined sets**		
18.	*7 AND 13 AND 17*	7,406
19.	*limit 18 to yr="2017 -Current"*	1,389
**Final result**		
**20.**		**1,389**

The final search result, usually found at the end of the documentation, forms the list of abstracts.

ti.ab,kf. = Title, abstract or Keyword Heading Word; **/** = Term from the Medline controlled vocabulary, but does not include terms (if existing) found below this term in the MeSH hierarchy; ***** = Truncation (if found at the end of a free text term); *adjN* = Positional operator that lets you retrieve records that contain your terms (in any order) within a specified number (n) of words of each other

Epistemonikos 13 March 2021

Search terms		Items found
**Age assessment**		
1.	(title:(((age OR matur* OR year*) AND (assess* OR chronologic* OR determ* OR estimat* OR evaluat* OR examin* OR forensic OR ident* OR legal OR measur* OR predict* OR verif*)) OR ((epiphys* OR "growth plate*") AND (adolescen* OR age OR child* OR matur* OR ossification OR year* OR "young adult*"))) OR abstract:(((age OR matur* OR year*) AND (assess* OR chronologic* OR determ* OR estimat* OR evaluat* OR examin* OR forensic OR ident* OR legal OR measur* OR predict* OR verif*)) OR ((epiphys* OR "growth plate*") AND (adolescen* OR age OR child* OR matur* OR ossification OR year* OR "young adult*"))))	
**Knee**		
2.	(title:(femur OR femoral OR knee* OR tibia*) OR abstract:(femur OR femoral OR knee* OR tibia*))	
**MRI**		
3.	(title:("magnetic resonance imag*" OR mr OR mri OR mris OR nmr OR radiolog* OR radiograp*) OR abstract:("magnetic resonance imag*" OR mr OR mri OR mris OR nmr OR radiolog* OR radiograp*))	
**Combined sets**		
4.	*1 AND 2 AND 3 (Year 2017–2021)*	198
**Final result**		
**5.**		**198**

The final search result, usually found at the end of the documentation, forms the list of abstracts.

* = Truncation

KSR Evidence 13 March 2021

Search terms		Items found
**Age assessment**		
1.	((age OR matur* OR year*) AND (assess* OR chronologic* OR determ* OR estimat* OR evaluat* OR examin* OR forensic OR ident* OR legal OR measur* OR predict* OR verif*)) OR ((epiphys* OR "growth plate*") AND (adolescen* OR age OR child* OR matur* OR ossification OR year* OR "young adult*"))	
**Knee**		
2.	(femur OR femoral OR knee* OR tibia*)	
**MRI**		
3.	("magnetic resonance imag*" OR mr OR mri OR mris OR nmr OR radiolog* OR radiograp*)	
**Combined sets**		
4.	*1 AND 2 AND 3 (Year 2017–2021)*	193
**Final result**		
**5.**		**193**

The final search result, usually found at the end of the documentation, forms the list of abstracts.

All text = All fields; ***** = Truncation; " " = Citation Marks; searches for an exact phrase

International HTA Database 13 March 2021

Search terms		Items found
**Age assessment**		
1.	((age OR matur* OR year*) AND (assess* OR chronologic* OR determ* OR estimat* OR evaluat* OR examin* OR forensic OR ident* OR legal OR measur* OR predict* OR verif*)) OR ((epiphys* OR "growth plate*") AND (adolescen* OR age OR child* OR matur* OR ossification OR year* OR "young adult*"))	
**Knee**		
2.	(femur OR femoral OR knee* OR tibia*)	
**MRI**		
3.	("magnetic resonance imag*" OR mr OR mri OR mris OR nmr OR radiolog* OR radiograp*)	
**Combined sets**		
4.	*1 AND 2 AND 3 (Year 2017–2021)*	0
5.		
**Final result**		
**6.**		**0**

The final search result, usually found at the end of the documentation, forms the list of abstracts.

All = All fields; ***** = Truncation; **" "** = Citation Marks; searches for an exact phrase

Supplement2 Excluded studies

Reference	Main reason for exclusion
Allen H, Liu F, Kijowski R, Nguyen J. T2mappingofarticular cartilageof the normal pediatric knee at 3.0 t. Skeletal Radiology. 2018;47(3):453–4.	Other reason
Boeyer ME, Ousley SD. Skeletal assessment and secular changes in knee development: a radiographic approach. American journal of physical anthropology. 2017;162(2):229–40.	Irrelevant population
Dallora AL, Berglund JS, Brogren M, Kvist O, Diaz Ruiz S, Dubbel A, et al. Age Assessment of Youth and Young Adults Using Magnetic Resonance Imaging of the Knee: A Deep Learning Approach. JMIR medical informatics. 2019;7(4) :e16291.	Other reason
Dallora AL, Kvist O, Berglund JS, Ruiz SD, Boldt M, Flodmark CE, et al. Chronological Age Assessment in Young Individuals Using Bone Age Assessment Staging and Nonradiological Aspects: Machine Learning Multifactorial Approach. JMIR medical informatics. 2020;8(9) :e18846.	Other reason
Ding KY, Dahlberg PS, Rolseth V, Mosdøl A, Straumann GH, Bleka Ø, et al. Development stages of the knee and ankle by computed tomography and magnetic resonance imaging for estimation of chronological age: a systematic review. 2018:60.	Irrelevant study design
Ekizoglu O, Hocaoglu E, Can IO, Inci E, Aksoy S, Bilgili MG. Magnetic resonance imaging of distal tibia and calcaneus for forensic age estimation in living individuals. International Journal of Legal Medicine. 2015;129(4):825-31. Available from: https://doi.org/10.1007/s00414-015-1187-1.	Irrelevant index test
Gräwert S. Forensic age assessment by means of MRI of the knee. RoFo Fortschritte auf dem Gebiet der Rontgenstrahlen und der Bildgebenden Verfahren. 2019;191(3):187–8.	Irrelevant language
Konigsberg LW, Sgheiza V. The Use of Roche, Wainer, and Thissen's Skeletal Maturity of the Knee. Journal of forensic sciences. 2019;64(6):1769-75.	Irrelevant index test
Kvist O, Luiza Dallora A, Nilsson O, Anderberg P, Sanmartin Berglund J, Flodmark CE, et al. A cross-sectional magnetic resonance imaging study of	Irrelevant population, partly

factors influencing growth plate closure in adolescents and young adults. Acta paediatrica (Oslo, Norway: 1992). 2021;110(4):1249–56.	Overlap with Kvist et al. 2021
Laor T, Chun GF, Dardzinski BJ, Bean JA, Witte DP. Posterior distal femoral and proximal tibial metaphyseal stripes at MR imaging in children and young adults. Radiology. 2002;224(3):669–74. Available from: https://doi.org/10.1148/radiol.2243011259.	Other reason
Maggio A. The skeletal age estimation potential of the knee: Current scholarship and future directions for research. Journal of Forensic Radiology and Imaging. 2017; 9:13-5.	Irrelevant study design
Mauer MAd, Well EJv, Herrmann J, Groth M, Morlock MM, Maas R, et al. Automated age estimation of young individuals based on 3D knee MRI using deep learning. International journal of legal medicine. 2021;135(2):649-63.	Other reason
Meza B, LaValva S, Aoyama J, DeFrancesco C, Striano B, Shea K, et al. Knee bone age using MRI: Validation of a novel method to reduce hand bone age radiographs. Orthopaedic Journal of Sports Medicine. 2020;8(7).	Other reason
Meza BC, LaValva SM, DeFrancesco CJ, Striano BM, Aoyama JT, Carey JL, et al. MRI knee bone age: A novel shorthand approach to reduce bone age radiographs in children. Orthopaedic Journal of Sports Medicine. 2020;8(4).	Other reason
Mostad P, Tamsen F. Error rates for unvalidated medical age assessment procedures. International journal of legal medicine. 2019;133(2):613-23.	Irrelevant study design
Nagrale N, Patond S, Ambad R, Bankar N, Jain K. Forensic age estimation from proximal end of femur: A radiological study in living individuals. Indian Journal of Forensic Medicine and Toxicology. 2020;14(4):7117-20.	Irrelevant index test
Pennock AT, Bomar JD. Bone age assessment utilizing knee MRI. Orthopaedic Journal of Sports Medicine. 2017;5(7).	Other reason
Pennock AT, Bomar JD, Manning JD. The Creation and Validation of a Knee Bone Age Atlas Utilizing MRI. The Journal of bone and joint surgery American volume. 2018;100(4): e20.	Irrelevant outcome

Prove PL, Jopp-van Well E, Stanczus B, Morlock MM, Herrmann J, Groth M, et al. Automated segmentation of the knee for age assessment in 3D MR images using convolutional neural networks. International journal of legal medicine. 2019;133(4):1191–205.	Other reason
Tamsen F. Results of age determinations indicate errors in the method. Resultat av aldersbedomningar pekar pa felaktigheter i metoden. 2017;114.	Irrelevant study design
Tamsen F. A majority of girls near the age of 18 may be misjudged as adults with MRI-knee. En majoritet av flickor nara 18 ar kan felbedomas som vuxna med MR-kna. 2017;114.	Irrelevant study design
Tamsen F. A majority of girls near the age of 18 may be misjudged as adults with MRI-knee. En majoritet av flickor nara 18 ar kan felbedomas som vuxna med MR-kna. 2017;114.	Duplicate
Timme M, Karch A, Shay D, Ottow C, Schmeling A. The relevance of body mass index in forensic age assessment of living individuals: an age-adjusted linear regression analysis using multivariable fractional polynomials. International journal of legal medicine. 2020;134(5):1861-8.	Irrelevant outcome
Timme M, Karch A, Shay D, Ottow C, Schmeling A. Age assessment of living individuals: the influence of socioeconomic status on skeletal and dental development in a German study cohort. Rechtsmedizin. 2021;31(1):35–41.	Other reason

Studies with high risk of bias

Ekizoglu O, Hocaoglu E, Inci E, Can IO, Aksoy S, Kazimoglu C. Forensic age estimation via 3-T magnetic resonance imaging of ossification of the proximal tibial and distal femoral epiphyses: Use of a T2-weighted fast spin-echo technique. Forensic Sci Int. 2016; 260:102. e1-. e7. Available from: https://doi.org/10.1016/j.forsciint.2015.12.006.

El-Din EAA, Mostafa HES, Tantawy EF, El-Shafei DA. Magnetic resonance imaging of the proximal tibial epiphysis: could it be helpful in forensic age estimation? Forensic science, medicine, and pathology. 2019;15(3):352–61.

Herrmann J, Saring D, Auf der Mauer M, Groth M, Jopp-van Well E. Forensic age assessment of the knee: proposal of a new classification system using two-dimensional ultrasound volumes and comparison to MRI. European radiology. 2020.

Supplement 3. QUADAS 2

Author: **Year**

Assessor:

Extra domains

**Item**	**Yes**	**Unclear**	**No**
Is the MR protocol well described?			
Age Mimicry: even age distribution?			

Domain 1. PATIENT SELECTION

A. Risk for bias

Describe methods of patient selection:

**Item**	**Yes**	**Unclear**	**No**
Was the selection process well described?			
Did the study avoid in inappropriate inclusions?			
Did the study avoid inappropriate exclusions?			
**Risk of bias**	low	moderate	high
**Could the selection of patients have introduced bias?**			

Domain 2. INDEX TEST

Describe the index test and how it was conducted and interpreted:

A. Risk for bias

**Item**	**Yes**	**Unclear**	**No**
Were the index test results interpreted without knowledge of the results of the reference standard?			
Was the ossification stage/classification well described ?			
Was the index test assessed by more than one observer?			
b) was the majority of images assessed by more than 2 observes			
**Risk of bias**	low	moderate	high
**Could the conduct of interpretation of the index test have introduced bias?**			

Domain 4. FLOW AND TIMING

A. Risk for bias

**Item**	**Yes**	**Unclear**	**No**
Were all patients included in the analysis?			
Risk of bias	low	moderate	high
**Could the patient flow have introduced bias?**			

Supplement 4 Characteristics of included studies

**Author**<br>**Year**<br>**Country**<br>**Ref nr**	Alatas<br>2021<br>Turkey<br>[1]
**Study design**	Retrospective cross-sectional cohort study
**Setting**<br>**Time period**	Radiology Clinic, Dokuz Eylül University, Turkey<br>January 2008- April 2018
**Population**<br><br><br>**Ethnicity**<br>**Age, sex**<br>**Sample**	Clinical population. Patients with knee pathology, chemotherapy, radiotherapy, steroid treatment or systemic/neoplastic disorders excluded.<br>Ethnicity not stated<br>Age: 12.01-27.55 years<br>N=709<br>Male: n=425<br>Female: n=284
**Ossification classifications:**	Vieth (2-6). Femur, tibia.
**Indextest**<br>**Tesla**<br>**Weight plan**<br>**Scan parameters**	**Field strength:** 1.5T<br>**Weight:** PD fs (spair) tse, T1 tse<br>**Plane**: Coronal<br>**Scan parameters**: PD: 3.5mm section thickness, coronal. TR 3400ms, TE 30 ms, duration 2:07min, FOV 512, matrix 256× 128 and NEX 1<br>T1: 4T1WI 3.5mm section thickness, coronal TR 480ms, TE 10ms, duration 1:22min, FOV 512, matrix256× 128 and NEX 1<br>**Resolution:** 2x4x3.5 mm
**No of observers**<br>**Intra/inter reliability**	Two observers, both experienced in forensic age estimation. 150 randomly selected scans examined together, the remaining examined separately. 100 scans were re-examined after 2 months. Examiners blinded to age of subjects.<br>Intra-observer reliability, K 0.828<br>Inter-observer reliability, K 0.841
**Outcome**	**Stages of distal femoral epiphysis**<br>*Males*: mean age (± SD); min-max age per age<br>Stage 2: 13.68 (1.29); 12.02-16.28<br>Stage 3: 15.07 (1.19); 12.34-18.92<br>Stage 4: 18.35 (1.99); 14.84-21.96<br>Stage 5: 21.02 (2.54); 15.81-26.71<br>Stage 6: 24.09 (1.73); 20.76-27.37<br>*Females*: mean age (± SD); min-max age per age<br>Stage 2: 12.89 (0.72); 12.01-14.53<br>Stage 3: 14.25 (1.11); 12.01-17.22<br>Stage 4: 16.26 (1.24); 13.77-19.08<br>Stage 5: 19.83 (2.83); 14.77-25.61<br>Stage 6: 23.89 (2.19); 20.45-27.55
**Comments**	Also includes measurements of proximal tibia.<br>Moderate risk of bias

**Author**<br>**Year**<br>**Country**<br>**Ref nr**	Altinsoy<br>2020<br>Turkey<br>[2]

**Study design**	Retrospective cross-sectional cohort study
**Setting**<br>**Time period**	Radiology Clinic, Elazig Training and Research Hospital, Turkey<br>January 2014 to December 2016
**Population**<br><br><br>**Ethnicity**<br>**Age, sex**<br>**Sample**	Clinical population. Patients with knee pathology, chemotherapy, radiotherapy, steroid treatment or systemic/neoplastic disorders excluded.<br>Ethnicity not stated.<br>Age:10-30 years<br>N=472<br>Male: n=277<br>Female: n=195
**Ossification classifications:**	Dedouit (1-5) Femur, Tibia
**Indextest**<br>**Tesla**<br>**Weight plan**<br>**Scan parameters**	Field strength: 1.5 T<br>Weight: PD fast SE<br>Plane: Coronal<br>Scan parameters: 4.5 mm section thickness, coronal TR: 3400 ms, TE: 30 ms, FOV: 250 × 200, matrix: 256 × 128, NEX: 1.<br>Resolution: 0.9x1.6x4.5mm
**No of observers**<br>**Intra/inter reliability**	Two observers, both radiologists evaluated all scans. 100 randomly selected scans were re-examined after 2 weeks.<br>Examiners blinded to age of subjects.<br>Intra-observer reliability, K 0.881/0.870 (observer 1/2)<br>Inter-observer reliability, K 0.759
**Outcome**	Stages of distal femoral epiphysis<br>Males: mean age (± SD); min-max age per stage<br>Stage 1: 13.42 (2.32); 10.23-16.70<br>Stage 2: 15.30 (1.65); 12.73-18.51<br>Stage 3: 19.80 (2.52); 14.94-26.70<br>Stage 4: 22.70 (3.19); 17.17-30.10<br>Stage 5: 25.72 (2.38); 21.83-30.98<br>Females: mean age (± SD); min-max age per stage<br>Stage 1: 12.30 (1.62); 10.26-14.03<br>Stage 2: 14.21 (1.37); 11.48-16.09<br>Stage 3: 17.36 (2.14); 13.43-22.39<br>Stage 4: 21.86 (3.54); 16.31-30.48<br>Stage 5: 25.14 (2.16); 21.23-29.68
**Comments**	Also includes measurements of proximal tibia.<br>Low risk of bias

**Author**<br>**Year**<br>**Country**<br>**Ref nr**	Auf der Mauer<br>2019<br>Germany<br>[3]
**Study design**	Prospective longitudinal cohort study
**Setting**<br>**Time period**	University Medical Center Hamburg-Eppendorf (UKE), Germany<br><br>Recruited April 2015- June 2017, followed with 3 scans over 2 years.
**Population**<br><br>**Ethnicity**<br>**Age, sex**<br>**Sample**	Healthy volunteers. Subjects with knee pathology or systemic disorders excluded.<br>Ethnicity not stated.<br>Age:14-19 years<br>N=40<br>Male: n=40<br>Female: n=0

**Ossification classifications:**	Jopp (1-3) Femur, Tibia, Fibula
**Indextest**<br>**Tesla**<br>**Weight plan**<br>**Scan parameters**	Field strength: 3T<br>Weight: T1 sense<br>Plane: coronal<br>Scan parameters: TR 850ms, TE 10ms, flip angle 90◦, resolution 800×800×41; in-plane resolution 0.1875×0.1875mmx 2; slice thickness 2mm; spacing between slices 2.2mm<br>Resolution: 0.1875×0.187x 2 mm
**No of observers**<br>**Intra/inter reliability**	Three observers, scientists in the field of forensic medicine. Blinded to age. Re-evaluation of all scans at follow-up.<br>Intra-observer reliability not stated<br>Inter-observer reliability, K 0.799 (femur)
**Outcome**	Majority of subjects scanned three times: baseline (BL), follow-up 1 (FU1) and follow-up 2 (FU2). The time gap between each MRI examination was 11 months on average (8-14 months).<br>Stages of distal femoral epiphysis, BL<br>Males: min-max age per stage<br>Stage 1: 14.4-17.8<br>Stage 2: 15.3-19.2<br>Stage 3: 16.3-21.7
**Comments**	Also includes measurements of proximal tibia and proximal fibula as well as calculations of SKJ, an overall score of the knee joint.<br>Moderate risk for bias

**Author**<br>**Year**<br>**Country**<br>**Ref nr**	Daghighi<br>2021<br>Iran<br>[4]
**Study design**	Retrospective cross-sectional cohort study
**Setting**<br>**Time period**	Tertiary hospital outpatient clinics<br>April 2016-April 2019
**Population**<br><br><br>**Ethnicity**<br>**Age, sex**<br>**Sample**	Patients referred to hospital for imaging of the knee for legal purposes. Patients with knee pathology, chemotherapy or corticosteroids excluded.<br>Ethnicity: Caucasoid race and Iranid type<br>Age:14-40 years (inclusion criteria 15-40 year)<br>N=193<br>Male: n=139<br>Female: n=54
**Ossification classifications:**	Schmelling (1-5), Femur, Tibia
**Indextest**<br>**Tesla**<br>**Weight plan**<br>**Scan parameters**	Field strength: 1.5 T<br>Weight: PD fs and T2 tse<br>Plane: Sagittal and coronal<br>Scan parameters: proton density fat sat: TR 2500 ms, TE 39 ms, slice thickness 4 mm, time for each acquisition: 2 min and 20 s, T2 sagittal: TR 4000 ms, TE 71 ms, slice thickness 4 mm, time for each acquisition: 2 min10s<br>Resolution: Not stated, not possible to calculate from given parameters
**No of observers**<br>**Intra/inter reliability**	Two observers, radiologists with at least 10 years of experience. All scans examined by both separately and re-examined after 30 days. Observers blinded to age, sex and name of subjects.<br>Intra-observer reliability, K 0.89/0.861 (each observer separately)<br>Inter-observer reliability, K 0.83
**Outcome**	Stages of distal femoral epiphysis

	Males: mean age (± SD)<br>Stage 1: 15.18 (0.603)<br>Stage 2: 16.56 (1.094)<br>Stage 3: 21.47 (5.137)<br>Stage 4: 29.08 (5.592)<br>Stage 5: 37.00 (4.243)<br>Females: mean age (± SD)<br>Stage 1: 15.00 (0.000)<br>Stage 2: 15.25 (0.500)<br>Stage 3: 16.43 (0.976)<br>Stage 4: 29.93 (5.443)<br>Stage 5: 37.17 (2.250)
**Comments**	Also includes measurements of proximal tibia. Results did not include minimal and maximum age within each stage. Results did include statistical testing (ANOVA and Tukey test) of mean age between stages.<br>Moderate risk for bias

**Author**<br>**Year**<br>**Country**<br>**Ref nr**	Dedouit<br>2012<br>France<br>[5]
**Study design**	Retrospective cross-sectional cohort study
**Setting**<br>**Time period**	Radiology Department, Centre Hospitalier Universitaire Rangueil, Toulouse, France<br>Time period not specified
**Population**<br><br><br>**Ethnicity**<br>**Age, sex**<br>**Sample**	Clinical population. Patients with knee pathology, chemotherapy, radiotherapy, steroid treatment, or systemic/neoplastic disorders excluded.<br>Ethnicity not stated.<br>Age: 10.1-30.9 years<br>N=290<br>Male: n=138<br>Female: n=152
**Ossification classifications:**	New classification model (later called Dedouit (1-5)). Femur, tibia.
**Indextest**<br>**Tesla**<br>**Weight plan**<br>**Scan parameters**	Field strength: 1.5T<br>Weight: PD fast spin echo, images show fs<br>Plane: Sagittal and coronal<br>Scan parameters: TR 2500–4000ms, TE25–50 ms, slice thickness 3.5–4mm, time for each acquisition:4min, 23 images<br>Resolution: Not stated, not possible to calculate from given parameters
**No of observers**<br>**Intra/inter reliability**	Two examiners, one radiologist and one forensic pathologist. The radiologist re-examined the scans after 3 weeks. Examiners blinded to age and name of subjects.<br>Intra-observer reliability, K 0.96<br>Inter-observer reliability K 0.86
**Outcome**	Stages of distal femoral epiphysis<br>Males: mean age (± SD); min-max age per age<br>Stage 1: 12.9 (1.71); 10.3-16.1<br>Stage 2: 15.5 (1.76); 12.1-18.9<br>Stage 3: 19.9 (3.20); 14.8-25.7<br>Stage 4: 23.6 (3.08); 17.8-30.0<br>Stage 5: 27.6 (2.15); 22.6-30.8<br>Females: mean age (± SD); min-max age per age

	Stage 1: 11.7 (1.28); 10.1-13.6<br>Stage 2: 13.6 (1.29); 11.0-15.7<br>Stage 3: 18.0 (3.36); 13.6-25.1<br>Stage 4: 22.7 (3.68); 16.6-29.6<br>Stage 5: 27.9 (2.70); 22.1-30.9
**Comments**	Also includes measurements of proximal tibia.<br>Moderate risk for bias

**Author**<br>**Year**<br>**Country**<br>**Ref nr**	Ekizoglu<br>2021<br>Turkey<br>[6]
**Study design**	Retrospective cross-sectional cohort study
**Setting**<br>**Time period**	Izmir Tepecik Training and Research Hospital, Turkey<br>2016-2019
**Population**<br><br><br>**Ethnicity**<br>**Age, sex**<br>**Sample**	Clinical population, suspicion of trauma or pathology to the knee. All patients with knee pathology, neoplastic disorders or radiation/chemotherapy excluded.<br>Ethnicity not stated<br>Age: 10-30 years<br>N=649<br>Male: n=355<br>Female: n=314
**Ossification classifications:**	Schmeling (five stages) and Kellinghaus (subclasses added). Staging defined in plain radiography (Schmeling) and computed tomography (Kellinghaus) originally. Femur, tibia.
**Indextest**<br>**Tesla**<br>**Weight plan**<br>**Scan parameters**	Field strength: 1.5 T<br>Weight: T1 tse<br>Plane: Sagital<br>Scan parameters: TR 345 ms, TE 11 ms, slice thickness 1.5mm, FOV180, acquisition time 2.3min.<br>Resolution: 0.35x0.35x1.5mm
**No of observers**<br>**Intra/inter reliability**	Two examiners, one expert in legal medicine and one radiologist. Re-evaluation by both after 4 weeks. Not stated if examiners were blinded to age and sex of subjects.<br>Intra-observer reliability, K 0.924<br>Inter-observer reliability K 0.898
**Outcome**	Stages of distal femoral epiphysis<br>Males: mean age (± SD) per stage; min-max age per age<br>Stage 2c: 12.35 (1.53); 10.0-15.3<br>Stage 3a: 15.65 (1.41); 12.7-18.7<br>Stage 3b: 16.52 (0.78); 15.1-17.5<br>Stage 3c: 17.26 (1.42); 15.8-21.9<br>Stage 4: 23.91 (3.19); 17.0-29.8<br>Females: mean age (± SD) per stage; min-max age per age<br>Stage 2c: 11.21 (0.82); 10.1-12.9<br>Stage 3a: 13.91 (0.91); 12.8-15.9<br>Stage 3b: 15.32 (0.30); 15.1-15.8<br>Stage 3c: 16.27 (1.22); 14.6-18.8<br>Stage 4: 23.63 (3.96); 15.4-29.8
**Comments**	Also includes measurements of proximal tibia<br>Moderate risk for bias

**Author**	Gurses

**Year**<br>**Country**<br>**Ref nr**	2020<br>Turkey<br>[7]
**Study design**	Retrospective cross-sectional cohort study
**Setting**<br>**Time period**	Radiology Clinic, Duzce University, Turkey<br>Jan 2012- June 2019
**Population**<br><br><br>**Ethnicity**<br>**Age, sex**<br>**Sample**	Clinical population. Patients with knee pathology, chemotherapy, radiotherapy, steroid treatment or systemic/neoplastic disorders excluded.<br>Ethnicity not stated.<br>Age: 12-30 years<br>N=598<br>Male: n=367<br>Female: n=231
**Ossification classifications:**	Vieth (2-6). Femur, tibia.
**Indextest**<br>**Tesla**<br>**Weight plan**<br>**Scan parameters**	**Field strength:** 1.5T<br>**Weight:** PDfs (T2w), T1 tse<br>**Plane**: coronal<br>**Scan parameters**: Proton density (PD _tse cor _fs) sequence: 3.5 mm section thickness; coronal TR:2000 ms; TE16 ms; FOV 200 mm; matrix: 256 × 128; NEX 1.T1 W imaging: 3.0 mm section thickness; sagittal/coronal TR: 450 ms; TE: 12 ms; FOV:200 mm; matrix: 256 × 128; NEX 1.<br>**Resolution:** 0.78x1.56x3.5mm<br>NB! This is a PD sequence, not a T2
**No of observers**<br>**Intra/inter reliability**	Two observers with experience in forensic age estimation. The images of 100 patients re-evaluated after 2 mo. Examiners blinded to age of subjects.<br>Intra-observer reliability, K 0.834<br>Inter-observer reliability K 0.823
**Outcome**	**Stages of distal femoral epiphysis**<br>*Males*: mean age (± SD); min-max age per age<br>Stage 2: 13.65 (1.11); 12.08-15.33<br>Stage 3: 16.22 (1.83); 12.92-19.50<br>Stage 4: 18.33 (1.40); 15.08-20.67<br>Stage 5: 21.46 (2.74); 15.83-30.50<br>Stage 6: 25.41 (3.07); 20.58-30.92<br>*Females*: mean age (± SD); min-max age per age<br>Stage 2: 13.14 (0.86); 12.08-14.75<br>Stage 3: 14.58 (0.89); 12.92-16.08<br>Stage 4: 16.81 (1.36); 14.33-19.67<br>Stage 5: 20.46 (2.86); 14.75-29.42<br>Stage 6: 25.06 (2.98); 20.58-30.92
**Comments**	Also includes measurements of proximal tibia.<br>Moderate risk for bias

**Author**<br>**Year**<br>**Country**<br>**Ref nr**	Kramer<br>2014<br>Germany<br>[8]
**Study design**	Retrospective cross-sectional cohort study
**Setting**<br>**Time period**	Center of Modern Diagnostics (ZEMODI), Bremen, Germany<br>2010-2012
**Population**	Clinical population. All patients with systemic/neoplastic disorders or steroid/chemotherapy excluded.

**Ethnicity**<br>**Age, sex**<br>**Sample**	Ethnicity not stated<br>Age: 10-30 years<br>N= 290<br>Male: n= 166<br>Female: n= 124
**Ossification classifications:**	Schmeling et al (five stages) and Kellinghaus et al (subclasses added). Staging defined in plain radiography (Schmeling) and computed tomography (Kellinghaus) originally. Femur.
**Indextest**<br>**Tesla**<br>**Weight plan**<br>**Scan parameters**	**Field strength:** 3T<br>**Weight:** T1 tse<br>**Plane**: Sagittal<br>**Scan parameters**: TR 783 ms, TE 13 ms, matrix, 512 [90 %], FOV 180 mm, slice thickness,3.0 mm, FA160°; voxel size, 0.4×0.4×3.0 mm; scan time,1 min 57 s<br>**Resolution:** 0.4×0.4×3.0 mm
**No of observers**<br>**Intra/inter reliability**	One examiner, experience in musculoskeletal MRI diagnostics. In 30 cases re-evaluation by the same examiner after 3 months + additional examiner.<br>Examiners blinded to age and sex of subjects.<br>Intra-observer reliability, K 0.94<br>Inter-observer reliability, K 0.85
**Outcome**	**Stages of distal femoral epiphysis**<br>*Males*: mean age (± SD) per stage; min-max age per age<br>Stage 2c: 12.3 (1.7); 10.1-15.5<br>Stage 3a: 15.0 (1.7); 12.2-19.4<br>Stage 3b: 15.1 (0.1); 15.0-15.1<br>Stage 3c: 17.0 (1.2); 15.0-19.5<br>Stage 4: 24.9 (3.5); 18.3-30.8<br>*Females*: mean age (± SD) per stage; min-max age per age<br>Stage 2c: 11.8 (1.40); 10.1-13.4<br>Stage 3a: 13.8 (1.70); 11.4-18.4<br>Stage 3b: -<br>Stage 3c: 17.0 (0.7); 15.6-18.2<br>Stage 4: 24.3 (4.0); 16.2-30.8
**Comments**	Moderate risk for bias

**Author**<br>**Year**<br>**Country**<br>**Ref nr**	Kvist<br>2020<br>Sweden<br>[9]
**Study design**	Prospective cross-sectional cohort study
**Setting**<br>**Time period**	Karolinska University Hospital and Blekinge Tekniska Högskola Health Technology Research Lab<br>October 2017-April 2018
**Population**<br>**Ethnicity**<br>**Age, sex**<br>**Sample**	Healthy volunteers.<br>Born in Sweden but ethnicity not stated. Subjects with bilateral knee pathology, chronic diseases or long-term medication excluded.<br>Age: 14.0-21.5 years<br>N=395<br>Male: n=217<br>Female: n=178
**Ossification classifications:**	Modified version of Dedouit and Schmeling (five stages). Subclasses by Kellinghaus
**Indextest**	**Field strength:** 1.5 T

**Tesla**<br>**Weight plan**<br>**Scan parameters**	**Weight:** T1 fse & cartilage<br>**Plane**: Coronal and sagittal<br>**Scan parameters**: TE 460 to 600 ms; TE 20 to x46 ms; slice thickness, 3 mm. FOV 160x160 mm Matrix 256x256<br>**Resolution:** 0.62x0.62x3mm
**No of observers**<br>**Intra/inter reliability**	Two pediatric radiologists and two general radiologists evaluated all scans. In case of disagreement between the observers, a third experienced pediatric radiologist assessed the images. Cartilage sequences not evaluated by general radiologists. All observers blinded to age and gender. After 4 weeks, a pediatric radiologist re-evaluated all scans.<br>Intra-observer reliability: femur, T1W-TSE: K 0.65, femur, cartilage sequences: K 0.79<br>Inter-observer reliability for pediatric radiologists: femur, T1W-TSE: K 0.73, femur, cartilage sequences: K 0.86<br>Inter-observer reliability for general radiologists: femur, T1W-TSE: K 0.56.
**Outcome**	Stages of distal femoral epiphysis<br>Males: Min-max age per stage (only stated in full years), TW1-TSE; cartilage sequences<br>Stage 2: 14-15; -<br>Stage 3: 14-17; -<br>Stage 4a: 14-18; 14-16<br>Stage 4b: 15-19; 14-18<br>Stage 4c: 15-21; 14-19<br>Stage 5: 16-21; 16-21<br>Females: Min-max age per stage (only stated in full years), TW1-TSE; cartilage sequences<br>Stage 2: -; -<br>Stage 3: 14-15; -<br>Stage 4a: 14-17; 14-15<br>Stage 4b: 14-16; 14-17<br>Stage 4c: 14-21; 14-17<br>Stage 5: 15-21; 14-21
**Comments**	Study not designed to assess chronological estimations of age, but rather to evaluate the growth plates of the knee in a descriptive manner.<br>Also includes measurements of proximal tibia.<br>A re-classification with only stages 1-5 (no subclassification of stage 4) improved the inter-observer agreement for pediatric radiologists but not for general radiologists.<br>Moderate risk for bias

**Author**<br>**Year**<br>**Country**<br>**Ref nr**	Margalit<br>2019<br>USA<br>[10]
**Study design**	Retrospective cross-sectional cohort study
**Setting**<br>**Time period**	Department of Orthopaedic Surgery, The Johns Hopkins Hospital, Baltimore, USA<br>January 2004- January 2014
**Population**<br><br>**Ethnicity**<br>**Age, sex**	Clinical population. Patients with knee pathology or systemic disorders excluded.<br>Ethnicity not stated.<br>Age: 6-19 years

**Sample**	N=165<br>Male: n=98<br>Female: n=67
**Ossification classifications:**	Dedouit (1-5), eightlocations both Femur, Tibia, Fibula
**Indextest**<br>**Tesla**<br>**Weight plan**<br>**Scan parameters**	**Field strength:** 1.5 T or 3T<br>**Weight:** Intermediate FSE, spair<br>**Plane**: Coronal or sagittal<br>**Scan parameters**: TE 2500 to 5000 ms; TE 25 to 40 ms; slice thickness, 3.5 to 4 mm<br>NB Variable weighting, all PD but more or less towards T2<br>**Resolution:** Not stated, not possible to calculate from given parameters
**No of observers**<br>**Intra/inter reliability**	Two observers (orthopedic surgeons) made all measurements, blinded to age of patient. The same rater measured the same image 1 week after the first measurement.<br>Intra-observer reliability, K 0.85 (overall, all locations)<br>Inter-observer reliability, K 0.88 (overall, all locations)
**Outcome**	**Stages of distal femoral epiphysis**<br>*Males*: mean age (± SD) for medial, central, lateral.<br>Stage 1: M: 6.8 (0.4), C: 9.3 (2.0), L: 6.8 (0.4)<br>Stage 2: M: 12.4 (2.2), C: 12.2 (1.6), L: 12.4 (2.2)<br>Stage 3: M: 14.8 (1.3), C: 15.5 (1.5), L: 14.8 (1.4)<br>Stage 4: M: 16.4 (1.8), C: 16.3 (1.8), L: 16.1 (1.6)<br>Stage 5: M: 17.9 (1.2), C: 18.5 (0.6), L: 18.5 (1.3)<br>*Females*: mean age (± SD) for medial, central, lateral<br>Stage 1: M: 8.3 (2.2), C: 8.3 (2.2), L: 8.3 (2.2)<br>Stage 2: M: 8.5 (2.2), C: 8.5 (2.2), L: 8.5 (2.2)<br>Stage 3: M: 13.4 (1.9), C: 12.4 (1.1), L: 13.3 (1.7)<br>Stage 4: M: 14.0 (2.2), C: 14.3 (2.2), L: 14.6 (2.2)<br>Stage 5: M: 17.2 (1.2), C: 17.2 (1.2), L: 17.2 (1.2)
**Comments**	Minimal and maximum age per stage was not stated. The 8 different locations measured on each patient were: medial, lateral and central femur, medial, lateral and central tibia, fibula and inferior tibeal tubercle (ITT). No significant differences in mean chronological age were detected within each stage between the different locations.<br>Moderate risk for bias

**Author**<br>**Year**<br>**Country**<br>**Ref nr**	Ottow<br>2017<br>Germany<br>[11]
**Study design**	Prospective cross-sectional cohort study
**Setting**<br>**Time period**	Germany<br>May 2013- June 2015
**Population**<br>**Ethnicity**<br>**Age, sex**<br>**Sample**	Healthy volunteers<br>German nationality (ethnicity not further specified)<br>Age: 12-24 years<br>N=658<br>Male: n=325<br>Female: n=333
**Ossification classifications:**	Schmeling (five stages) and Kellinghaus (subclasses added). Staging defined in plain radiography (Schmeling) and computed tomography (Kellinghaus) originally. Femur, tibia.
**Indextest**<br>**Tesla**	Field strength: 3T<br>Weight: T1 TSE

**Weight plan**<br>**Scan parameters**	Plane: Coronal<br>Scan parameters: TR 633 ms, TE 20ms, flip angle 90, duration 3:51 min; measured voxel size 0.6 × 0.77 ×3 mm, reconstructed voxel size 0.31 × 0.31 × 3 mm<br>Resolution: 0.6 × 0.77 ×3 mm
**No of observers**<br>**Intra/inter reliability**	One examiner with experience in musculoskeletal MRI diagnostics. 115 randomly chosen cases were re-examined by same examiner after 2 mo +additional examiner. The examiners were blinded to age and sex of subjects.<br>Intra-observer reliability, K 0.961<br>Inter-observer reliability, K 0.941
**Outcome**	Stages of distal femoral epiphysis<br>Males: mean age (± SD) per stage; min-max age per age<br>Stage 2c: 14.12 (1.45); 12.05-19.15<br>Stage 3a: 15.95 (1.22); 13.68-17.88<br>Stage 3b: 17.77 (-); -<br>Stage 3c: 17.95 (2.12); 16.13-24.84<br>Stage 4: 21.5 (2.03); 17.46-24.98<br>Females: mean age (± SD) per stage; min-max age per age<br>Stage 2c: 13.42 (1.14); 12.11-15.74<br>Stage 3a: 14.80 (0.91); 13.39-17.82<br>Stage 3b: 15.89 (1.23); 14.73-19.5<br>Stage 3c: 16.21 (1.22); 14.53-20.62<br>Stage 4: 20.72 (2.38); 16.13-25.00
**Comments**	Also involves measurements of proximal tibia<br>Moderate risk for bias

**Author**<br>**Year**<br>**Country**<br>**Ref nr**	Uygun<br>2020<br>Turkey<br>[12]
**Study design**	Retrospective cross-sectional cohort study
**Setting**<br>**Time period**	Cukurova University Faculty of Medicine, Balcali Hospital, Adana, Turkey<br>January 2012- April 2018
**Population**<br><br><br>**Ethnicity**<br>**Age, sex**<br>**Sample**	Clinical population. Patients with knee pathology, chemotherapy, radiotherapy, steroid treatment or systemic/neoplastic disorders excluded.<br>Ethnicity not stated.<br>Age: 10-25 years<br>N=489<br>Male: n=292<br>Female: n=197
**Ossification classifications:**	Dedouit (1-5) Femur, Tibia
**Indextest**<br>**Tesla**<br>**Weight plan**<br>**Scan parameters**	Field strength: 1.5T<br>Weight: PD fs FSE<br>Plane: Coronal<br>Scan parameters: section thickness, 4 mm; TR, 2600ms; TE 42ms; FOV,170x170; slice, 20; Nex, 2.<br>Resolution: Not stated, not possible to calculate from given parameters
**No of observers**<br>**Intra/inter reliability**	Number of observers for all cases not stated. 100 randomly selected cases were re-evaluated after two weeks for intra-observer and inter-observer reliability. Observers were blinded to age and sex.<br>Intra-observer reliability, K 0.955

	Inter-observer reliability, K 0.913
**Outcome**	Stages of distal femoral epiphysis<br>Males: mean age (± SD); min-max age per stage<br>Stage 1: 12.24 (1.546); 10-16<br>Stage 2: 13.51 (1.487); 11-16<br>Stage 3: 17.96 (2.129); 14-25<br>Stage 4: 21.51 (2.029); 16-25<br>Stage 5: 23.57 (1.826); 15-25<br>Females: mean age (± SD); min-max age per stage<br>Stage 1: 11.67 (1.090); 10-13<br>Stage 2: 13.25 (1.597); 10-17<br>Stage 3: 16.78 (2.309); 12-23<br>Stage 4: 20.48 (2.227); 15-25<br>Stage 5: 23.37 (2.116); 14-25
**Comments**	Age stated in full years only. Also includes measurements of proximal tibia.<br>Moderate risk for bias

**Author**<br>**Year**<br>**Country**<br>**Ref nr**	Vieth<br>2018<br>Germany<br>[13]
**Study design**	Prospective cross-sectional cohort study
**Setting**<br>**Time period**	Germany<br>May 2013- June 2015
**Population**<br>**Ethnicity**<br>**Age, sex**<br>**Sample**	Healthy volunteers<br>German nationality (ethnicity not further specified)<br>Age: 12-24 years<br>N=694<br>Male: n=344,<br>Female: n=350
**Ossification classifications:**	New classification model (later called Vieth, 2-6). Femur, tibia.
**Indextest**<br>**Tesla**<br>**Weight plan**<br>**Scan parameters**	Field strength: 3T<br>Weight: T1 and T2 spir<br>Plane: Coronal<br>Scan parameters: T1 TR633 ms; TE 20 ms; flip angle 90 degree; duration 3:51 min<br>measured voxel size 0.6 × 0.77 × 3 mm; reconstructed voxel size 0.31 × 0.31 × 3 mm.<br>T2-w TSE SPIR TR shortest; TE 65 ms; flip angle 90 degree; duration 3:08 min; measured voxel size 0.6 × 0.76 × 3 mm; reconstructed voxel size 0.31 ×0.31 × 3 mm<br>Resolution: 0.6 × 0.76 × 3 mm
**No of observers**<br>**Intra/inter reliability**	One examiner with experience in musculoskeletal MRI diagnostics. 100 randomly chosen cases were re-examined by same examiner after 2 mo + additional examiner, experienced in musculoskeletal diagnostics. The examiners were blinded to age and sex of subjects.<br>Intra-observer reliability, K 0.914<br>Inter-observer reliability K 0.913
**Outcome**	Stages of distal femoral epiphysis<br>Males: mean age (± SD); min-max age per age<br>Stage 2: 13.39 (0.89); 12.05-15.56<br>Stage 3: 14.75 (1.66); 12.13-19.15<br>Stage 4: 17.04 (0.81); 15.49-18.81<br>Stage 5: 21.20 (2.23); 15.71-24.98

	Stage 6: 23.23 (1.32); 21.24-24.70<br>Females: mean age (± SD) per stage; min-max age per age<br>Stage 2: 12.41 (0.31); 12.11-12.88<br>Stage 3: 13.83 (1.02); 12.16-15.74<br>Stage 4: 15.90 (1.00); 14.33-18.46<br>Stage 5: 20.50 (2.54); 14.82-24.98<br>Stage 6: 22.62 (1.20); 20.65-24.05
**Comments**	Partly same population as in #6 but with different testing and different purpose of the study (new classification system).<br>Also includes measurements of proximal tibia.<br>Moderate risk for bias.

References

1. Alatas O, Altınsoy HB, Gurses MS, Balci A. Evaluation of knee ossification on 1.5 T magnetic resonance images using the method of Vieth et al.: A retrospective magnetic resonance imaging study. Rechtsmedizin. 2021;31(1):50-8.
2. Altinsoy HB, Alatas O, Gurses MS, Turkmen Inanir N. Forensic age estimation in living individuals by 1.5T magnetic resonance imaging of the knee: a retrospective MRI study. Australian Journal of Forensic Sciences. 2020;52(4):439-53.
3. Auf der Mauer M, Saring D, Stanczus B, Herrmann J, Groth M, Jopp-van Well E. A 2-year follow-up MRI study for the evaluation of an age estimation method based on knee bone development. International journal of legal medicine. 2019;133(1):205-15.
4. Daghighi MH, Pourisa M, Javanpour-Heravi H, Ghojazadeh M, Mirza-Aghazadeh-Attari M, Daghighi S, et al. Application of knee MRI in forensic age estimation: A retrospective cohort. Radiography (London, England : 1995). 2021;27(1):108-14.
5. Dedouit F, Auriol J, Rousseau H, Rouge D, Crubezy E, Telmon N. Age assessment by magnetic resonance imaging of the knee: a preliminary study. Forensic Sci Int. 2012;217(1-3):232.e1-7. Available from: https://doi.org/https://dx.doi.org/10.1016/j.forsciint.2011.11.013.
6. Ekizoglu O, Er A, Bozdag M, Basa CD, Kacmaz IE, Moghaddam N, et al. Forensic age estimation via magnetic resonance imaging of knee in the Turkish population: use of T1-TSE sequence. International journal of legal medicine. 2021;135(2):631-7.
7. Gurses MS, Altinsoy HB. Evaluation of distal femoral epiphysis and proximal tibial epiphysis ossification using the Vieth method in living individuals: applicability in the estimation of forensic age. Australian Journal of Forensic Sciences. 2020.
8. Kramer JA, Schmidt S, Jürgens KU, Lentschig M, Schmeling A, Vieth V. Forensic age estimation in living individuals using 3.0 T MRI of the distal femur. Int J Legal Med. 2014;128(3):509-14. Available from: https://doi.org/10.1007/s00414-014-0967-3.
9. Kvist OF, Dallora AL, Nilsson O, Anderberg P, Berglund JS, Flodmark CE, et al. Comparison of reliability of magnetic resonance imaging using cartilage and T1-weighted sequences in the assessment of the closure of the growth plates at the knee. Acta radiologica open. 2020;9(9):2058460120962732.
10. Margalit A, Cottrill E, Nhan D, Yu L, Tang X, Fritz J, et al. The Spatial Order of Physeal Maturation in the Normal Human Knee Using Magnetic Resonance Imaging. Journal of pediatric orthopedics. 2019;39(4):e318-e22.

11. Ottow C, Schulz R, Pfeiffer H, Heindel W, Schmeling A, Vieth V. Forensic age estimation by magnetic resonance imaging of the knee: the definite relevance in bony fusion of the distal femoral- and the proximal tibial epiphyses using closest-to-bone T1 TSE sequence. European radiology. 2017;27(12):5041-8.

12. Uygun B, Kaya K, Kose S, Ekizoglu O, Hilal A. Applicability of Magnetic Resonance Imaging of the Knee in Forensic Age Estimation. The American journal of forensic medicine and pathology. 2020.

13. Vieth V, Schulz R, Heindel W, Pfeiffer H, Buerke B, Schmeling A, et al. Forensic age assessment by 3.0T MRI of the knee: proposal of a new MRI classification of ossification stages. European radiology. 2018;28(8):3255-62.

Supplement 5 Risk of bias chart

Studies included in the systemativ review (low and moderate risk of bias)

green= low risk, yellow= moderate risk, red=high risk

Author	Is the MR protocol well described?	Age Mimicry: even age distribution?	Could the selection of patients have introduced bias?	Could the conduct or interpretation of the index text have introduced bias?	Could the patient flow have introduced bias?	Total bias	Comments
Alatas 2021	green	red	green	yellow	green	yellow	Minority of the image was evaluated by 2 observers
Altinsoy 2020	green	red	green	green	green	green	
Auf der Mauer 2019	green	red	yellow	green	green	yellow	
Daghighi 2021	yellow	red	green	green	green	yellow	
Dedouit 2012	yellow	red	green	green	green	yellow	
Ekizoglu 2021	green	red	green	yellow	green	yellow	Re-evaluation not blinded
Gurses 2020	green	red	green	green	green	yellow	
Krämer 2014	green	red	green	yellow	green	yellow	Minority of the image was evaluated by 2 observers
Kvist 2020	green	green	yellow	green	green	yellow	
Margalit 2019	yellow	red	green	green	yellow	yellow	
Ottow 2017	green	green	yellow	yellow	green	yellow	Minority of the image was evaluated by 2 observers,
Uygun 2020	green	red	green	yellow	green	yellow	Unclear how many of the images that had been evaluated by two observes,
Vieth 2018	green	green	yellow	yellow	green	yellow	Minority of the image was Evaluated by 2 observers

Supplement 6

PPV, NPV, Sensitivitet och Specificitet

PPV: the proportion of the populations with a mature knee and that is18 year or older?

NPV: the proportion of the population with a immature knee that is under the age of 18.

Sensitivity: the proportion of the 18 years of age that have a mature knee:

Specificity: the proportion of the populations that is under 18 years of age with an immature knee.

Tabell 3. *How PPV, NPV, sensitivity and specificity change depends on population tested (se table x in the article for data regarding the 5 hypothetical populations) .*

Study	Sex	age	PPV	NPV	Sensitivity	Specificity
	male	17 – 18	86.4 %	60.3 %	38.0 %	94.0 %
	male	16 – 19	94.7 %	71.7 %	62.0 %	96.5 %
Kramer	male	15 – 20	96.9 %	79.0 %	74.0 %	97.7 %
	male	15 – 18	84.4 %	82.5 %	38.0 %	97.7 %
	male	17 – 20	97.4 %	54.7 %	74.0 %	94.0 %
	female	17 – 18	61.2 %	67.1 %	74.0 %	53.0 %
	female	16 – 19	70.8 %	79.0 %	82.5 %	66.0 %
Kramer	female	15 – 20	77.5 %	85.5 %	87.3 %	74.7 %
	female	15 – 18	49.3 %	89.6 %	74.0 %	74.7 %
	female	17 – 20	84.8 %	58.2 %	87.3 %	53.0 %
	male	17 – 18	71.6 %	69.5 %	68.0 %	73.0 %
	male	16 – 19	82.9 %	80.7 %	80.0 %	83.5 %
Ottow	male	15 – 20	88.4 %	86.4 %	86.0 %	88.7 %
	male	15 – 18	66.7 %	89.3 %	68.0 %	88.7 %
	male	17 – 20	90.5 %	63.5 %	86.0 %	73.0 %
	female	17 – 18	54.3 %	80.0 %	95.0 %	20.0 %
	female	16 – 19	61.4 %	92.9 %	97.0 %	39.0 %
Ottow	female	15 – 20	68.7 %	96.5 %	98.0 %	55.3 %
	female	15 – 18	41.5 %	97.1 %	95.0 %	55.3 %
	female	17 – 20	78.6 %	76.9 %	98.0 %	20.0 %
	male	17 – 18	76.5 %	68.1 %	62.0 %	81.0 %
	male	16 – 19	87.5 %	79.5 %	77.0 %	89.0 %
Ekizoglu	male	15 – 20	92.0 %	85.5 %	84.3 %	92.7 %
	male	15 – 18	73.8 %	88.0 %	62.0 %	92.7 %
	male	17 – 20	93.0 %	63.3 %	84.3 %	81.0 %
	female	17 – 18	57.1 %	75.0 %	89.0 %	33.0 %
	female	16 – 19	64.8 %	87.6 %	93.0 %	49.5 %

Ekizoglu	female	15 – 20	71.6 %	92.6 %	95.0 %	62.3 %
	female	15 – 18	44.1 %	94.4 %	89.0 %	62.3 %
	female	17 – 20	81.0 %	68.8 %	95.0 %	33.0 %
	male	17 – 18	59.6 %	53.8 %	34.0 %	77.0 %
	male	16 – 19	68.1 %	57.7 %	40.5 %	81.0 %
Altinsoy	male	15 – 20	75.0 %	61.4 %	47.0 %	84.3 %
	male	15 – 18	42.0 %	79.3 %	34.0 %	84.3 %
	male	17 – 20	86.0 %	32.6 %	47.0 %	77.0 %
	female	17 – 18	59.3 %	57.8 %	54.0 %	63.0 %
	female	16 – 19	67.9 %	65.3 %	62.5 %	70.5 %
Altinsoy	female	15 – 20	74.6 %	71.3 %	69.3 %	76.3 %
	female	15 – 18	43.2 %	83.3 %	54.0 %	76.3 %
	female	17 – 20	84.9 %	40.6 %	69.3 %	63.0 %
	male	17 – 18	63.0 %	52.0 %	17.0 %	90.0 %
	male	16 – 19	72.9 %	54.0 %	21.5 %	92.0 %
Dedouit	male	15 – 20	80.2 %	56.1 %	27.0 %	93.3 %
	male	15 – 18	45.9 %	77.1 %	17.0 %	93.3 %
	male	17 – 20	89.0 %	29.1 %	27.0 %	90.0 %
	female	17 – 18	57.5 %	55.0 %	46.0 %	66.0 %
	female	16 – 19	64.4 %	59.9 %	52.5 %	71.0 %
Dedouit	female	15 – 20	70.4 %	64.6 %	58.7 %	75.3 %
	female	15 – 18	38.3 %	80.7 %	46.0 %	75.3 %
	female	17 – 20	83.8 %	34.7 %	58.7 %	66.0 %
	male	17 – 18	65.0 %	74.0 %	80.0 %	57.0 %
	male	16 – 19	75.8 %	85.2 %	87.5 %	72.0 %
Vieth	male	15 – 20	82.3 %	90.3 %	91.3 %	80.3 %
	male	15 – 18	57.6 %	92.3 %	80.0 %	80.3 %
	male	17 – 20	86.4 %	68.7 %	91.3 %	57.0 %
	female	17 – 18	53.0 %	82.4 %	97.0 %	14.0 %
	female	16 – 19	58.7 %	93.9 %	98.0 %	31.0 %
Vieth	female	15 – 20	65.6 %	97.3 %	98.7 %	48.3 %
	female	15 – 18	38.5 %	98.0 %	97.0 %	48.3 %
	female	17 – 20	77.5 %	77.8 %	98.7 %	14.0 %
	male	17 – 18	72.6 %	60.1 %	45.0 %	83.0 %
	male	16 – 19	84.6 %	69.3 %	60.5 %	89.0 %
Kvist	male	15 – 20	90.3 %	76.1 %	71.0 %	92.3 %
	male	15 – 18	66.2 %	83.4 %	45.0 %	92.3 %
	male	17 – 20	92.6 %	48.8 %	71.0 %	83.0 %
	female	17 – 18	60.5 %	65.4 %	72.0 %	53.0 %
	female	16 – 19	69.3 %	76.3 %	80.0 %	64.5 %
Kvist	female	15 – 20	75.9 %	83.0 %	85.0 %	73.0 %
	female	15 – 18	47.1 %	88.7 %	72.0 %	73.0 %
	female	17 – 20	84.4 %	54.1 %	85.0 %	53.0 %
	male	17 – 18	67.4 %	54.8 %	29.0 %	86.0 %
	male	16 – 19	78.8 %	59.5 %	39.0 %	89.5 %
Uygun	male	15 – 20	86.0 %	64.3 %	49.0 %	92.0 %

	male	15 – 18	54.7 %	79.5 %	29.0 %	92.0 %
	male	17 – 20	91.3 %	36.0 %	49.0 %	86.0 %
	female	17 – 18	62.3 %	56.5 %	43.0 %	74.0 %
	female	16 – 19	71.9 %	62.6 %	52.5 %	79.5 %
Uygun	female	15 – 20	79.1 %	68.1 %	60.7 %	84.0 %
	female	15 – 18	47.3 %	81.6 %	43.0 %	84.0 %
	female	17 – 20	87.5 %	38.5 %	60.7 %	74.0 %
	male	17 – 18	64.7 %	57.6 %	44.0 %	76.0 %
	male	16 – 19	75.9 %	64.7 %	55.0 %	82.5 %
Gurses	male	15 – 20	82.8 %	70.8 %	64.3 %	86.7 %
	male	15 – 18	52.4 %	82.3 %	44.0 %	86.7 %
	male	17 – 20	88.9 %	41.5 %	64.3 %	76.0 %
	female	17 – 18	57.6 %	67.2 %	80.0 %	41.0 %
	female	16 – 19	64.8 %	78.7 %	85.5 %	53.5 %
Gurses	female	15 – 20	71.1 %	85.7 %	89.3 %	63.7 %
	female	15 – 18	42.3 %	90.5 %	80.0 %	63.7 %
	female	17 – 20	82.0 %	56.2 %	89.3 %	41.0 %
